# Plant Virus-Like Particle In Situ Vaccine for Intracranial Glioma Immunotherapy

**DOI:** 10.3390/cancers11040515

**Published:** 2019-04-10

**Authors:** Amber Kerstetter-Fogle, Sourabh Shukla, Chao Wang, Veronique Beiss, Peggy L. R. Harris, Andrew E. Sloan, Nicole F. Steinmetz

**Affiliations:** 1 Department of Neurological Surgery, Case Western Reserve University, 10900 Euclid Avenue, Cleveland, OH 44106, USA; aek20@case.edu (A.K.-F.); plr2@case.edu (P.L.R.H.); 2 Department of NanoEngineering, University of California San Diego, La Jolla, CA 92093, USA; sshukla@ucsd.edu (S.S.); chw022@eng.ucsd.edu (C.W.); vbeiss@eng.ucsd.edu (V.B.); 3 University Hospitals-Cleveland Medical Center & the Seidman Cancer Center, Case Western Reserve University, 10900 Euclid Avenue, Cleveland, OH 44106, USA; 4 Case Comprehensive Cancer Center, Case Western Reserve University, 10900 Euclid Avenue, Cleveland, OH 44106, USA; 5 Department of Radiology, University of California San Diego, La Jolla, CA 92093, USA; 6 Department of Bioengineering, University of California San Diego, La Jolla, CA 92093, USA; 7 Moores Cancer Center, University of California San Diego, La Jolla, CA 92093, USA

**Keywords:** intracranial glioma, immunotherapy, CPMV, viral nanoparticles, in situ vaccine

## Abstract

Despite aggressive multi-modality treatment with surgery, radiation and chemotherapies, malignant glioma inevitably recurs and has dismal survival rates. Recent progress in immunotherapy has led to a resurgence of interest, and immunotherapies are being investigated for treatment of glioma. However, the unique brain anatomy and a highly immunosuppressive glioma microenvironment pose significant challenges to achieving efficacy. Thus, there is a critical need for assessment of next-generation immunotherapies for glioma. In this study, we have investigated the efficacy of the nanoparticle platform technology based on plant-derived Cowpea mosaic virus like particles (empty CPMV or eCPMV) to instigate a potent immune response against intracranial glioma. CPMV immunotherapy has been shown to efficiently reverse the immunosuppressive tumor microenvironments in pre-clinical murine models of dermal melanoma and metastatic melanoma, metastatic breast cancer, intraperitoneal ovarian cancer and in canine patients with oral melanoma. In the present study, we demonstrate that in situ administration of CPMV immunotherapy in the setting of glioma can effectively recruit unique subset of effector innate and adaptive immune cells to the brain parenchyma while reducing immune suppressive cellular population, leading to regression of intracranial glioma. The in situ CPMV nanoparticle vaccine offers a potent yet safe and localized immunotherapy for intracranial glioma.

## 1. Introduction

Malignant glioma represents one of the most aggressive forms of cancer, with poor survival rates that have not changed in the past three decades despite advancements in detection and treatment modalities. Even with aggressive treatments including debulking, chemotherapy and radiation the median survival rates for malignant glioma is 12 months, with a five-year relative survival of about 5% [1,2]. Glioma is also associated with high rates of morbidity due to damage to functional regions of the brain. Surgery, radiation and chemotherapy are the mainstay of treatment regimens [3]. Malignant glioma is incurable surgically due to the infiltrative nature of these tumors and the surgeon’s inability to safely resect a “margin” as is typical in most other solid cancers. The blood brain barrier (BBB) also limits the penetration and clinical efficacy of most systemic chemotherapies [4]. Moreover, residual tumor subpopulations resistant to radio- and chemotherapy eventually lead to recurrence and treatment failure [5,6].

Success of immunotherapies for other solid tumors has led to renewed interest in immunotherapy for gliomas [7,8]. However, the unique anatomical and physiological features of the brain are critical barriers for such interventions [9]. The BBB, blood-cerebrospinal fluid (CSF) and blood-meningeal barriers limit the entry of most small molecules as well as immune cells into the brain [10]. Recent advances in the understanding of the central nervous system (CNS) have however, reversed the longstanding theory that the brain is “immunologically privileged” and demonstrated that the BBB in patients with malignant glioma is typically disrupted [11]. It has recently been noted that activated T cells can cross the blood-brain-barrier, however, many of the inflammatory cells recruited to malignant gliomas contribute to the highly immunosuppressive tumor microenvironment (TME). The glioma TME is characterized by the prevalence of M2 polarized resident macrophages and microglial cells, myeloid-derived suppressor cells (MDSCs), and a significantly smaller population of antigen presenting cells (APCs) including exhausted dendritic cells (DCs) and T cells. The expression of vascular endothelial growth factor (VEGF), tumor growth factor-β (TGF-β), prostaglandin E2 (PGE2) and interleukin-10 (IL-10) further impair T cell proliferation and activation in response to pro-inflammatory signals, and downregulate expression of major histocompatibility complexes (MHCs) and DC maturation [12]. Malignant glioma also has a considerable infiltration of regulatory T cells (Tregs), which can further suppress proliferation and activation of tumor infiltrating T lymphocytes [13]. Additionally, glioma stem cells (GSCs) and glioma initiating cells (GIC), which drive glioma growth and invasion, contribute to the immunosuppressive tumor microenvironment by recruiting tumor-supportive macrophages and microglia, expressing immunosuppressive cytokines, and down regulating Toll-like receptors (TLRs) thereby avoiding immune-mediated rejection [14].

Several immunotherapeutic approaches targeting gliomas are currently under pre-clinical development and clinical evaluation. These include vaccines, adoptive T cell therapies, monoclonal antibodies, checkpoint inhibitors and oncolytic virotherapies [8]. Immune escape with loss of targeted antigens and glioma heterogeneity pose significant challenges for vaccines based on single or multiple antigens, respectively [15]. Additionally, lack of a robust population of resident APCs in the brain contributes to the reduced efficacy of glioma vaccines [12]. DC vaccines can overcome this hurdle by reintroducing the patient’s own antigen pulsed DCs, however besides the technological challenges, this approach suffers from low efficacy attributed to poor migration of DCs to secondary lymphoid tissues for T cell activation [16]. Adoptive cell therapies (ACT) [17] that can circumvent DC dependence have proven to be challenging in malignant gliomas due to their low densities within tumors, tumor mediated exhaustion, and inefficient delivery through intravenous administrations [8,9]. Similarly, restrictive delivery through the BBB and lack of effector cells or cytotoxic T cells (CTLs) renders antibody drug conjugates and checkpoint inhibitors less effective as brain tumor treatments [18,19].

Oncolytic virotherapy utilizing genetically engineered mammalian viruses to selectively invade glioma cells and express cytotoxic proteins has emerged as a potent therapeutic strategy for glioma. Several oncolytic viruses have been studied including adenoviral vector Ad-RTS-hIL-12 that expresses IL12 in the presence of an orally administered ligand, which can efficiently cross the BBB to reach the tumor bed [20]. Similarly, engineered poliovirus PVSRIPO [21], oncolytic herpes simplex virus type-1 (oHSV-1) [22], measles virus [23] and Zika virus [24] have been shown to selectively replicate in and lyse CNS tumor cells or tumor stem cells. While highly effective, applicability of virotherapy to the brain remains under investigation, as uncontrolled inflammatory responses posing a threat to healthy brain cells and delivery to the brain remain major hurdles [9].

Here, we present data from an in situ vaccine immunotherapy against malignant glioma utilizing the plant virus like particles (VLPs) derived from Cowpea mosaic virus (CPMV). The CPMV VLPs lacks their nucleic acids and are referred to as empty CPMV (eCPMV). Recently, we have shown that eCPMV (as well as CPMV) can induce anti-tumor responses in several murine models of cancers when introduced into the tumor microenvironment (TME) as an in situ vaccine [25]. Results from canine trials have also demonstrated efficacy in large animals with spontaneous melanoma [26]. Mechanistic insights have indicated that CPMV stimulates an anti-tumor response through recruitment of innate cells including monocytes, tumor infiltrating neutrophils (TINs) and natural killer (NK) cells, which exert cancer cell toxicity resulting in the release of tumor antigens. Furthermore, through elevated influx of APCs into the treated tumors, the CPMV in situ vaccine also facilitates priming of an adaptive anti-tumor response with CD4^+^/CD8^+^ cells, therefore leading to systemic efficacy and immunological memory [25]. Thus, CPMV activates the innate immune response, recalibrating the cancer–immunity cycle to eliminate cancer cells via the adaptive immune system. Unlike the oncolytic viruses, plant viruses are non-infectious and non-replicative in mammalian cells and the immune modulation is driven by the unique proteinaceous architecture of the viral capsid. While previously validated in various mouse model of dermal and metastatic melanoma, lung metastasis from breast cancer, and intraperitoneal ovarian cancer, in the present study we set out to assess the efficacy of eCPMV in situ vaccine against intracranial glioma using the syngeneic GL261 glioma mouse model.

## 2. Results

**Figure 1:**
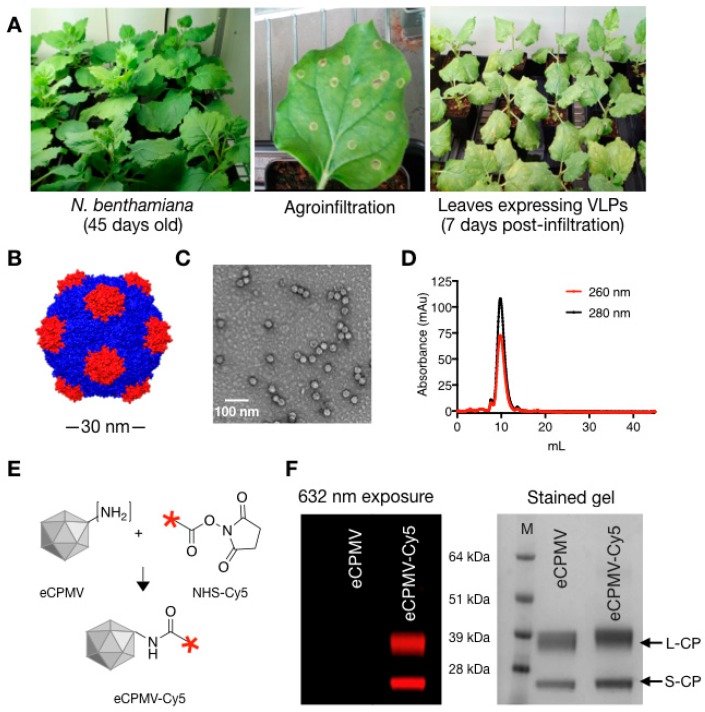
eCPMV propagation, purification and characterization. (**A**) eCPMV VLPs were propagated using *N. benthamiana* plants; leaves were infiltrated when plants were 45 days old. (**B**) Structure of eCPMV, chimera image created using PDB file 1NY7 (CPMV) (**C**,**D**) Purified eCPMV particles were characterized for structural integrity using TEM and size exclusion chromatography (FPLC). (**E**) One-step-NHS chemistry was used to bioconjugate Cy5 dyes to the lysine residues on eCPMV CPs. (**F**) SDS-gel electrophoresis was used to confirm conjugation of Cy5 dyes to eCPMV coat proteins: the fluorescence derived from conjugated Cy5 is detected by exposing the gel to 632 nm excitation; the small (S) and large (L) protein are detected after protein staining (GelCode™ Blue Safe protein stain) and visualization under white light.

CPMV VLPs were produced by co-expression of the precursor to the L and S coat proteins (VP60) and the viral proteinase (24K) using *N. benthamiana* plants and agroinfiltration as previously described (Figure 1A) [27]. Typical purification yields are around 0.5 mg of VLP from each gram of leaf tissue. The eCPMV VLP is a 30 nm-sized icosahedral particle devoid of nucleic acid and consists of 60 copies each of a small (S) and large (L) coat protein subunits (Figure 1B) [28]. Post-purification quality assurance of the self-assembled VLP was performed using TEM imaging and size exclusion chromatography (SEC) confirming the presence of intact 30 nm particles with the typical elution profiles from the Superose column; the absorbance ratio of A260:280 of 0.67 indicates that particles devoid of RNA were produced (in contrast RNA-containing wild-type CPMV has a A260:280 of 1.8 [29] (Figure 1C,D). For imaging and tracking studies, *N*-hydroxysuccinimide chemistry was used to conjugate the NHS esters of sulfo-Cy5 fluorophores to eCPMV capsid via the exposed lysine residues [30] (Figure 1E). SDS-gel electrophoresis was used to confirm fluorescent tagging of the viral coat proteins (Figure 1F). Unmodified eCPMV is represented by two distinct bands corresponding to the ~24 kDa small coat protein (S-CP) subunit and the ~42 kDa large coat protein subunit (L-CP). In eCPMV-Cy5 particles both these protein bands appear fluorescent when excited at 632 nm, indicating successful dye conjugation. The ratio of Cy5 dyes per eCPMV particle were quantified by UV/Vis spectroscopy and using the particles’ specific extinction coefficient ε_eCPMV_ of 1.28 mL mg^−1^ cm^−1^ at 280 nm and the molar extinction coefficient ε_Cy5_ of 271,000 at 660 nm. The reaction yielded ~50 dyes per eCPMV.

To assess the potential for development of CPMV immunotherapy for treatment of glioma, we first determined the feasibility of delivering VLP immunotherapy through intracranial injections into the brain using fluorescently labeled eCPMV-Cy5 VLPs. All mouse studies were performed in compliance with the Institutional Animal Care and Use Committee of Case Western Reserve University.

**Figure 2:**
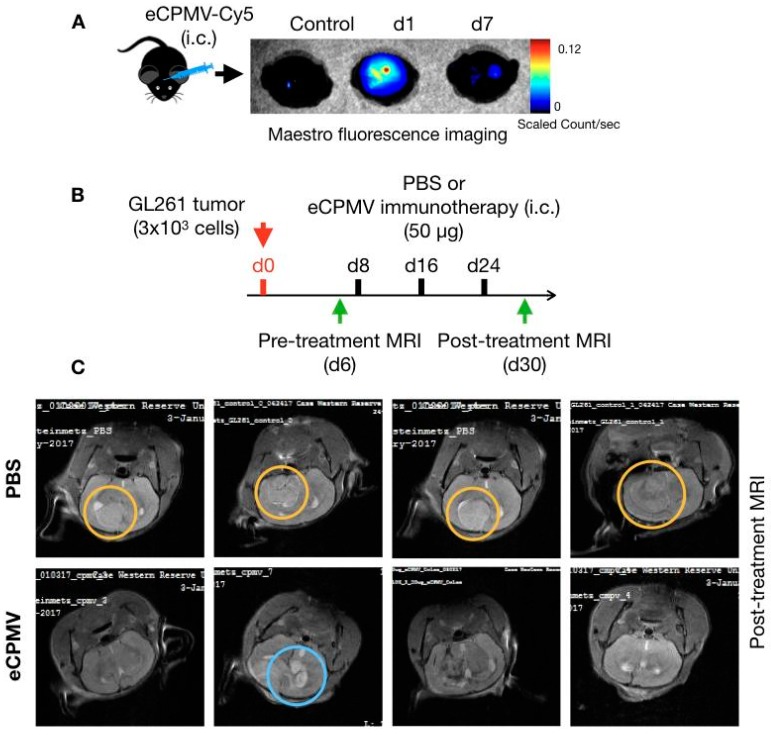
Intracranial eCPMV injection and immunotherapy. (**A**) eCPMV retention in brain following intracranial administration was determined using eCPMV-Cy5 and Maestro fluorescence imaging system. (**B**) For in situ immunotherapy, C57BL6 mice (*n* = 4) were inoculated with 3 × 10^3^ GL261 cells intracranially and administered PBS or eCPMV via intracranial injections on days 8, 16 and 24. (**C**) On day 30, MRI imaging (7 Tesla) was used to visualize glioma post-treatment. Yellow circles highlight solid tumors in PBS administered mice, whereas blue circle highlights the residual tumor and/or edema in one of the mice in the eCPMV treatment group.

Following the intracranial injection, presence and retention of VLPs in brain was detected using ex vivo Maestro fluorescence imaging (Figure 2A). At 24 h post injection eCPMV can be readily detected at and around the injection site as evident by a strong fluorescent signal, whereas at day 7 the weak signal intensity suggested degradation and loss of proteinaceous VLPs from the brain microenvironment (Figure 2A). This observation is consistent with clearance of viral nanoparticles from other tissues [31]. During this period, mice were monitored for any signs of stress and discomfort resulting from particle administration and no apparent adverse effects were observed. We also evaluated intravenous route for eCPMV VLP administration as a mean of delivery immunotherapy to the brain. However, the VLPs were sequestered in the liver and spleen by the mononuclear phagocyte system (MPS) system with minimal doses reaching other tissues, including the brain. Therefore, we selected the intracranial injection as the mode of delivery for our studies. Based on the weeklong retention of eCPMV in brain, we set weekly intratumoral administration schedules for glioma immunotherapy.

Next, we assessed the immunotherapeutic potential of the eCPMV in situ vaccine in a mouse model of GL261 glioma. This syngeneic model based on immunocompetent mice is one of the most widely used animal models for gliomas [32]. C57BL6 mice were challenged with syngeneic GL261 tumors via intracranial injections of 3 × 10^3^ cells in 3 μL of PBS. Pre-treatment MRI was performed on day 6 post-tumor inoculations to establish onset of tumor growth; then mice were randomly assigned to treatment groups (Figure 2B). eCPMV immunotherapy at a dose of 50 μg VLP in 3 μL sterilized PBS was administered via weekly intracranial injections starting on day 8 post tumor inoculation. The control group received sterile PBS. Mice were observed for signs associated with glioma progression including weight loss, irregular breathing, hunched back and decreased activity; upon appearance of these signs, mice were euthanized. Following three intracranial injections, a second MRI was performed on day 30 post-tumor inoculations to assess treatment efficacy (Figure 2B).

Representative brain MRI images from PBS or eCPMV treated mice highlighted the therapeutic effects of the eCPMV in situ vaccination. Untreated mice developed large intracranial tumors by day thirty following the tumor inoculation (Figure 2C, upper panel; area marked with orange circle). In some mice, peritumoral edema was also observed as bright spots at the tumor edges. These mice also displayed the characteristic neurological and physiological symptoms associated with glioma including hunched posture, lack of activity and labored breathing. In stark contrast, eCPMV-treated mice showed absence of tumors or appeared to have regressed intracranial tumors (Figure 2C, lower panel) suggesting a therapeutic effect of the intratumoral eCPMV administrations. Presence of cerebral edema (blue circle) indicates stimulation of an inflammatory response to the in situ eCPMV.

**Figure 3:**
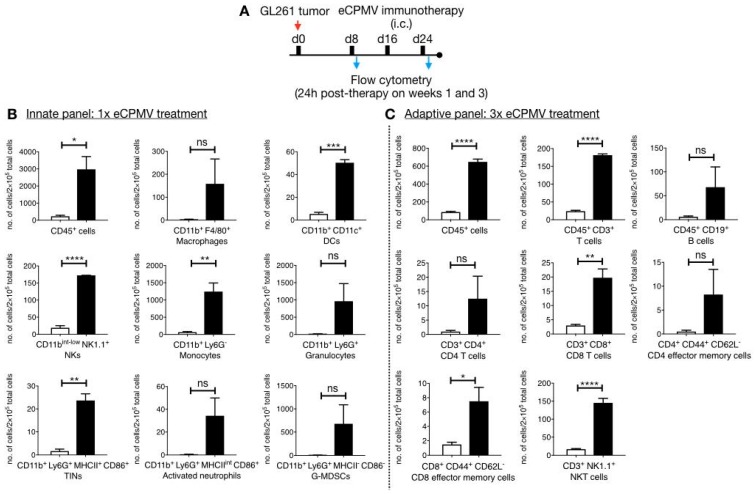
Flow cytometry analysis to characterize immune infiltration in the brain parenchyma. (**A**) GL261 glioma bearing C57BL6 mice (*n* = 3) were treated 1× or 3× with eCPMV immunotherapy and the brain tissues were harvested 24 h following the last treatments to determine innate (**B**) and adaptive (**C**) immune cell infiltrates in eCPMV treated (black bars) or untreated (white bars) GL261 bearing brain tissues. Error bars represent SEM. Statistical comparisons were performed using unpaired *t*-test (**** *p* < 0.0001, *** *p* < 0.001, ** *p* < 0.01 and * *p* < 0.05).

To validate the underlying immunology of the eCPMV in situ vaccine, we used flow cytometry and IHC analysis. For flow cytometry, brains were harvested from immunized mice 24 hours post single or three weekly eCPMV administrations (Figure 3A). The single cell suspension from brains that received a single dose of CPMV VLPs were used for characterization of the innate immune response (Figure 3A) whereas tissues and cells derived from animals that received multiple eCPMV doses were used to characterize the adaptive immune response (Figure 3B). Flow cytometry analysis after a single intracranial injection indicates enhanced immune cells infiltration into the brain of mice receiving eCPMV in situ vaccine (black bars) over tumor bearing mice receiving PBS (white bars). Specifically, eCPMV treated mice showed significantly elevated levels of leukocytes (CD45^+^ cells), CD11b^+^CD11c^+^ DCs, CD11b^int-low^NK1.1^+^ NK cells, CD11b^+^Ly6G^−^ monocytes, and CD11b^+^Ly6G^+^MHCII^+^CD86^+^ TINs compared with PBS-treated tumor bearing mice. Several other immune cells including macrophages, granulocytes, activated neutrophils and G-MDSCs showed an increasing trend, but were not statistically significantly different from PBS treated mice.

The innate response to the in situ vaccine is consistent with our earlier studies using mouse models of melanoma [33]. Here, we observed monocytes being recruited; in particular elevated levels of CD11b^+^Ly6G^−^ monocytes in eCPMV-treated brain could suggest local inflammation in response to the intracranial injection of the VLPs. Further, both monocytic CD11b^+^CD11c^+^ DCs and CD11b^+^F4/80^+^ macrophages were elevated in response to eCPMV immunotherapy. Additionally, eCPMV treatment also resulted in significant influx of the CD11b^+^Ly6G^+^MHCII^+^CD86^+^ tumor infiltrating neutrophils (TINs) that also displayed high expression levels of CD86 and MHCII molecules (Figure 3B).

Next, brain tissues harvested from mice following three treatments of eCPMV or PBS were analyzed by flow cytometry to evaluate the role of adaptive immunity (Figure 3C). Unlike the innate response, tumor-specific CD8^+^ and CD4^+^ T cells proliferate in an antigen-specific manner following stimulation by APCs. The potent innate response generated by the eCPMV in situ vaccine indeed led to significant recruitment of CD3^+^ T cells. Specifically, CD8^+^ T cell levels were significantly higher in eCPMV-treated mice over PBS-treated tumor-bearing mice. The increase in the CD4^+^ T cell population was not significantly different comparing eCPMV-vs PBS-treated tumor-bearing mice. A similar trend was observed for the effector memory T cells (EMTs), where a significant increase in the levels of CD8^+^ CD44^+^ CD62L^−^ EMTs and a non-significant increase in the levels of CD4^+^CD44^+^CD62L^−^ EMTs was noted comparing eCPMV- vs. PBS-treated animals. Interestingly, eCPMV treated mice also demonstrated significantly enhanced levels of CD3^+^NK1.1^+^ NKT cells. Overall, these results demonstrate efficient recruitment of both innate and adaptive immune cells to the brain tissue as a result of the eCPMV in situ vaccination, indicating that the anti-tumor response is indeed immune-mediated.

**Figure 4:**
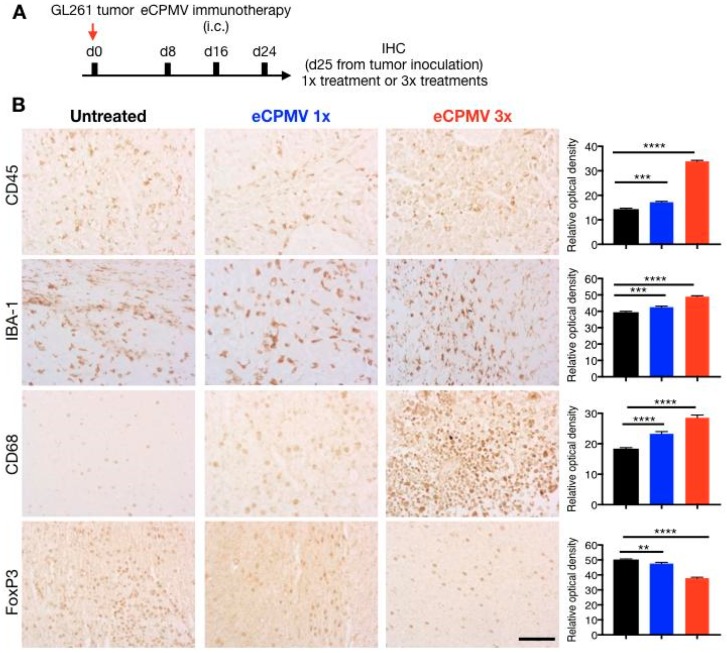
Immunohistochemical analysis. (**A**) Brain tissues from GL261 glioma bearing mice (*n* = 3) were harvested on day 25 from tumor inoculation after 24 h of receiving 1× eCPMV treatment or the last dose of 3× eCPMV treatment. (**B**) Tumor sections (6 μm thick) were stained with α-CD45 antibody, α-IBA-1 antibody, α-CD68 antibody and α-FoxP3 antibody. The scale bar is 50 μm in all images. Quantitative analysis was performed using Zeiss software to determine relative optical densities of the stained sections. Error bars represent SEM. Statistical analysis was performed using ordinary one-way ANOVA (Tukey’s multiple comparison test (**** *p* < 0.0001, *** *p* < 0.001, ** *p <* 0.01).

Finally, we used immunohistochemistry (IHC) to characterize the changing cellular landscape of the innate immune system within the glioma itself. To gauge the effects of single or multiple doses of eCPMV, brains from tumor-bearing mice were harvested 24 h after either a single dose or three doses (Figure 4A). Fixed tissue sections were then stained for immune cell markers IBA-1, CD68, CD45 and FoxP3 and cellular densities were quantified using Zeiss image analysis program (Axiovision Rel 4.5, Zeiss, Thornwood, NY, USA) (Figure 4B). IHC staining revealed significantly elevated levels of CD45^+^ leucocytes in the tumor tissue following the eCPMV treatment, which mirrors the flow cytometry data indicating an overall increase in the CD45^+^ cells in the brain parenchyma. When compared to a single administration, the overall CD45^+^ optical density doubled following three doses of eCPMV in situ vaccine; this may indicate engagement of adaptive cell response that results in an influx of effector cells including T lymphocytes and NKT cells. Further, we quantified the changes in IBA-1 and CD68 expression to evaluate microglia/macrophage (IBA-1/CD68) invasion into the tumors. As observed in IHC panel (Figure 4B), eCPMV administration resulted in increased intratumoral expression levels of both IBA-1 and CD68. We also compared the population of Tregs in glioma bearing mice with and without CPMV immunotherapy by staining for FoxP3 expression, which is a regulatory T cells specific transcription factor [34]. Our results indicate abundance of FoxP3^+^ Tregs in non-treated glioma, whereas a significant reduction in intratumoral FoxP3^+^ Tregs is observed with CPMV immunotherapy (Figure 4B).

## 3. Discussion

We and others have demonstrated that intratumorally administered VLPs can modulate the local tumor microenvironment via recruitment and activation of immune cells [25,35]. The immunomodulatory properties of VLPs arise from the repetitive architectures of the coat proteins that present potent pathogen-associated molecular patterns (PAMPs) [36]. VLPs are recognized by TLRs [37] and TLR signaling can stimulate innate as well as adaptive immune responses. Previously, GL261 cells have also been shown to express TLR2, TLR3 and TLR4, and TLR ligands have been used as treatments against established glioma [38,39]. Here we show that intratumoral administration of the eCPMV VLP generates an antitumor immune response leading to immunological regression of glioma. In some cases, inflammatory responses were also noted. The formation of such cerebral edema induced by inflammatory response has been previously observed following glioma radiotherapy [40] and oncolytic viral therapies [41]. The risk management of the immune responses in the brain as part of the immunotherapy will require future detailed investigation.

Profiling of the innate and adaptive immune cells following the in situ vaccination provides insight into the mechanism. Consistent with our previous studies in other tumor models, the innate immune cell cohort consists of monocytic DCs and macrophages and TINs. TINs have been identified as the primary modulators of the anti-tumor innate responses. Direct physical contact between neutrophils and cancer cells has been shown as a pre-requisite for cancer cell cytotoxicity. The cytotoxicity is attributed to neutrophil-secreted H_2_O_2_ that induces influx of Ca^2+^ in cancer cells leading to apoptosis [42] or to neutrophil Fas ligand-cancer cell Fas receptor interactions, which stalls tumor cell cycle progression from G1 to S phase [43]. In addition to direct cytotoxicity, neutrophils also mediate anti-tumor immune response through recruitment of effector immune cells. Pro-inflammatory N1 neutrophils promote CD8^+^ T recruitment and activation by secreting chemokines (e.g., CCL3, CXCL9, and CXCL10) and cytokines (e.g., IL-12, TNF-α, GM-CSF) that attract T cells [44]. Moreover, neutrophils can also coordinate adaptive immune responses through interactions with dendritic cells [44].

Another significant population of infiltrating innate immune cells was identified as NK cells. NK cells are among the most potent cytotoxic cells against tumor cells and high levels of tumor infiltrating NK cells are associated with a favorable tumor outcome in patients [45]. Transformed cells with reduced or absent MHC-I expression are therefore NK cells targets. Additionally, cellular stress and DNA damage results in upregulation of NK cell activation ligands on tumors. NK cells can kill tumor cells by releasing cytolytic granules containing perforin and granzymes, which leads to cancer cell apoptosis; NK cells also induce death receptor-mediated apoptosis [45,46]. Furthermore, activated NK cells secrete IFN-γ that can induce CD8^+^ T cells to CTL transformation and promote CD4^+^ T cells towards Th1 response, which promotes CTL differentiation. NK cells also promote recruitment of conventional DC-type 1 (cDCs) in the tumor microenvironment via secreted CCL5 and XCL1 cytokines [47]. cDCs are particularly efficient in carrying tumor antigens and cross presenting them to CD8 T cells to stimulate an adaptive immune response [48]. Therefore, tumor antigens released from cells lysed by activated NK cells can be taken up by APCs including the DCs and TINs described above, which subsequently can contribute towards development of an adaptive immune response mediated by tumor-specific CTLs [45].

Recent advances in the understanding of the structural and functional aspects of CNS lymphatic vessels have revealed the mechanism of CNS immune surveillance, including the entrance and exit of immune cells [11]. Functional studies of such meningeal lymphatic vessels have revealed their role in transporting CD11c^+^ cells, B220^+^ cells and T cells. Additionally, these meningeal lymphatic vessels have been shown connected to deep cervical lymph nodes, which are known to elicit immune responses to antigens from cerebrospinal fluids [49,50]. Thus, APCs carrying tumor antigens from lysed cells following eCPMV treatment are likely transported to the draining deep cervical lymph nodes and present the tumor antigens to naïve T cells resulting in expansion of tumor antigen specific adaptive immune response.

Indeed, our data indicate engagement of cells of the adaptive immune system. We note a significant increase in CD8^+^ T cells levels, effector memory CD8 T cells and NKT cells. NKTs are a unique lineage of innate T cells that express markers for T lymphocytes as well as NK cells. These cells can kill tumor cells by direct cytolysis using perforin and granzyme B, but also by modulating the recruitment of other effector immune including T cells, B cells, NK cells and DCs [51]. Thus, NKT cells may also play a key role in linking the innate and adaptive immune response against tumors. NKT cells can also reverse the immunosuppression mediated by MDSCs and tumor associated macrophages [52]. In the case of glioma that display highly immunosuppressive tumor microenvironment, the role of NKT as boosters of adaptive immune response and suppressor of immune regulation is therefore considered critical.

Furthermore, IHC highlighted the changing immunological landscape following in situ vaccination with eCPMV. Our data indicate significantly elevated levels of CD45^+^ cells in the brain paranchyma following CPMV administration, which corroborates earlier studies in melanoma models where intratumoral administrations of CPMV lead to massive influx of effector immune cells [33]. In particular, we observed enhanced influx of IBA-1/CD68^+^ microglia/macrophage cells. Microglia are the resident innate immune cells of CNS that participate in immune surveillance and host defense against infectious agents. Together with infiltrating bone marrow-derived macrophages, microglia functions to restore homeostasis in brain parenchyma to counter inflammatory responses including malignancies. The dual role of microglia/macrophages in gliomas has been extensively studied. Under immunosuppressive TME, microglia/macrophages appear to promote glioma proliferation and invasiveness [53,54]. However, microglia-secreted factors and TLR agonists have been shown to simulate apoptosis in glioma cells [55,56]. Also, while genetic ablation of monocytoid cells has been shown to promote glioma, drug mediated activation of monocytoid cells in brain results in microglia-mediated reduction of brain tumor initiating cells [57]. This functional duality has been attributed to the polarization of microglia to tumor suppressive M1 and tumor supportive M2 cells [58,59]. M1 cells are activated by type I cytokines such as interferon-γ (IFN-γ) and tumor necrosis factor-α (TNF-α) and other immunostimulants such as lipopolysaccharide (LPS), and lipoproteins. The activated M1 polarized microglia have been shown to possess antigen-presenting capabilities to Th1 cells leading to anti-tumor CTL activity [58]. CPMV has been shown to stimulate IFN-γ and TNF-α in the TME in our previous studies, and therefore is likely to influence the glioma TME similarly. These results are consistent with elevated staining observed in glioma treated with LPS or myeloid cell activation agents that also lead to increased iNOS expression (M1 phenotype) and a significant reduction in tumor volumes [57].

Tumor-mediated immune suppression is a critical barrier to glioma immunotherapies. In addition to M2 polarized microglia/macrophages described above, regulatory T cells (Tregs) accumulated in the TME contribute to glioblastoma-mediated immune suppression [60]. In high grade brain tumors, Tregs suppress activation, proliferation and cytokine production of CD4^+^/CD8^+^ T cells via secreted cytokines such as TGF-B and IL-10 or via cell-to cell contact mediated by the constitutively expressed CTLA-4 and PD-L1 checkpoints [61]. Thus, expansion of Tregs is associated with decreased efficacy of immunotherapies and therapeutic targeting of Tregs has been used to improve survival in glioma studies [62,63]. The significant reduction in intratumoral FoxP3^+^ Tregs following CPMV immunotherapy mirrors the effects of intratumoral IL-12^+^ CTLA-4 combination therapy which enhanced infiltration of CD4/CD8 cells while significantly reducing the FoxP3^+^ Tregs [64]. Pro-inflammatory cytokines including IL-12 are key component of eCPMV-mediated immune response and likely contribute to reduced Tregs population in the glioma TME [25,33]. Overall, eCPMV immunotherapy leads to an effective reversal of the immunosuppressive tumor microenvironment. In conjugation with the elevated CD8^+^ T cells and NKT cells infiltration and enhanced activation of resident microglia, this immunomodulation renders the glioma TME conducive for progression of an anti-tumor immune response.

## 4. Materials and Methods

### 4.1. Production of eCPMV VLPs

eCPMV VLP was produced as described elsewhere [25,27]. Briefly, Agrobacterium LBA4404 cultures harboring the binary plasmid pEAQexpress-VP60-24K that encodes the coat protein precursor VP60 and viral proteinase 24K, were introduced into *N. benthamiana* leaves using syringe-infiltration. Infiltrated tissue was harvested 6 days post-infiltration, homogenized in 0.1 M sodium phosphate buffer (pH 7.0) and purified using established protocols [27]. VLP concentration was determined by UV/vis spectroscopy (ε_280 nm_ = 1.28 mg^−1^ mL cm^−1^). Particle integrity was examined using transmission electron microscopy (TEM) on a FEI Technai20 and by size exclusion chromatography using a Superose 6 column on the AKTA Explorer chromatography system (GE Healthcare, Chicago, IL, USA).

### 4.2. Synthesis and Characterization of eCPMV-Cy5 Particles

eCPMV VLPs were covalently modified with Cy5 using N-hydroxysuccinimide-activated ester targeting surface exposed lysine residues on the capsid. Briefly, 3000 molar excess of Sulfo-Cyanine5 NHS ester (NHS-Sulfo Cy5) were reacted with eCPMV in 0.1M KP buffer at final protein concentration of 2 mg/mL in presence of 10% (*v*/*v*) DMSO. Following overnight reactions, eCPMV-Cy5 was purified from unconjugated reactants over a 40% (*w*/*v*) sucrose cushion at 160,000× *g* for 3 h and resuspended in sterile KP buffer. UV spectroscopy was used to determine the eCPMV-Cy5 concentrations and to determine Cy5/VLPs ratios using molar extinction coefficient ε_eCPMV_ of 1.28 mL mg^−1^ cm^−1^ at 280 nm and sulfo-Cy5-specific molar extinction coefficient ε_Cy5_ of 271,000 at 660 nm.

The conjugation of fluorophore on eCPMV was determined using SDS-gel electrophoresis. Briefly, 10 μg of unmodified eCPMV and eCPMV-Cy5 mixed with SDS running buffer and heated at 100 °C for 5 min were loaded on pre-cast NuPAGE™ 4–12% Bis-Tris proteins gels (ThermoFisher Scientific, Hampton, NH, USA) and electrophoresis was performed for 40 min at 200 V. Fluorescent bands representing Cy5 modified eCPMV coat proteins were visualized on an AlphaImage gel documentation system (Protein simple) using a 632 nm excitation. The gels were then stained using GelCode™ Blue Safe protein stain (ThermoFisher Scientific).

### 4.3. Cell Line

GL261 cell line was obtained from the Tumor Repository at National Cancer Institute (NCI) and maintained in suspension culture prior to engraftment intracranially. Briefly, cells were grown in a suspension flask (CytoOne, CC-672-4175, USA Scientific, Oscala, FL, USA) and kept in a 5% CO_2_ 37 °C humidified incubator in serum-free neuro medium (MACs neuro medium with Neurobrew-21 (130-093-570 and 130-097-263 respectively, Miltenyi Biotec Inc., Auburn, CA, USA), 20 ng/mL EGF (AF-100-15, Peprotech, Rocky Hill, NJ, USA) and 20 ng/mL FGF (100-18B, Peprotech) with 1% (*w*/*v*) pencillin-streptomycin (15140122, Gibco Invitrogen, Waltham, MA, USA) and 1% (*w*/*v*) L-glutamine (25030081, Gibco Invitrogen). Cultured cells were pelleted and re-suspended in media to 3 × 10^3^ cells per 3 μL in growth medium and placed on ice prior to implantation.

### 4.4. Tumor Inoculation

All mouse studies were performed in compliance with the Institutional Animal Care and Use Committee of Case Western Reserve University (Assurance number is A-3145-01, valid until 20 April 2019). Immunocompetent animals (C57BL6, Jackson Labs, Bar Harbor, MA, USA), 4–6 weeks of age, males and females, were utilized for intracranial implantation of GL261 cells (*n* = 4). Briefly, animals were placed under anesthesia (inhaled isoflurane). Once fully anesthetized, lidocaine was applied and a small incision was made through the scalp and the bregma was identified. A small 25-gauge burr hole was made 2 mm caudal and 3 mm to the right of bregma. A 22-gauge Hamilton syringe (88011, ThermoFisher Scientific) was inserted and placed 3 mm below the skull and then retracted 0.5 mm to establish a pocket for implantation of cells. Cells, 3 × 10^3^ cells per 3 μL PBS, were slowly injected into the right frontal lobe and the Hamilton syringe was held in place for 3 min post injection to prevent reflux. The burr hole was sealed with bone wax and the incision was closed with surgical glue and non-dissolvable sutures. Animals were given analgesia and maintained on a heating pad until recovery. Control and CPMV treated animals were placed under anesthesia weekly and given intratumoral injections of either vehicle or CPMV (50 μg) in 3 μL dosages. Injections were made within the same burr hole as done with inoculation of tumor. This was performed weekly for a total of 3 weeks.

Care and housing of the animals was provided by the University Animal Resource Center following IACUC oversight. The facility follows recommendations from the Guide for the Care and Use of Laboratory Animals of the National Institutes of Health. Mice were maintained in microisolator cages and exposed to 12 h light/12 h darkness cycles with standard food and water *ad libitum*. Mice were weighed weekly and checked daily for tumor growth symptoms according to the IACUC tumor burden policy.

### 4.5. Small Animal MRI

MRI imaging was performed prior to start of immunotherapy at day 6 from tumor inoculation and post-treatment on day 30. The in vivo MRI studies were performed on the same Biospec 7 T scanner equipped with a 3 cm birdcage ^1^H coil (Bruker, Erlangen, Germany). During MR imaging, mice were anesthetized by isoflurane, respiration rate was maintained at 70–80/min. After reaching surgical plane of anesthesia, the mouse was placed on an animal holder with its nose inserted into a nose cone. A head restrainer was utilized to prevent potential motion. An animal monitoring system was in place to monitor body temperature and respiration/cardiac cycle. After securing the animal and monitoring the components, the animal was positioned at the center of the RF coil. The RF coil was placed into the magnet. We conducted shimming process using a single pulse sequence and the RF pulse was maximized to keep the pulse length constant and a long enough recycle delay to conduct an image. A fast image acquisition was used to acquire sample images to determine animal placement and imaging setup.

### 4.6. Flow Cytometry

The following antibodies and reagents were used for flow cytometry, all obtained from BioLegend (San Diego, CA, USA): Pacific Blue anti-mouse CD45 (clone 30-F11), FITC anti-mouse CD11b (clone M1/70), APC anti-mouse CD11c (clone N418), PE anti-mouse F4/80 (clone BM8), Brilliant Violet 605 anti-mouse CD86 (clone GL-1), Alexa Fluro 700 anti-mouse I-A/I-E (clone M5/114.15.2), APC/Cy7 anti-mouse CD3 (clone 145-2C11), FITC anti-mouse CD4 (clone Gk1.5), APC anti-mouse CD8 (clone 53-6.7), Alexa Fluor 700 anti-mouse CD25 (clone PC61), PE anti-mouse FOXP3 (clone MF-14), Zombie yellow fixable viability kit, and anti-mouse CD16/32 (clone 93). GL261 Glioma bearing mice (*n* = 3) were treated once or thrice with CPMV immunotherapy or PBS and brain tissues were harvested 24 h following the intratumoral therapy. Single-cell suspensions were prepared as previously described [25] and incubated for 15 min at 4 °C with a CD16/CD32 antibody (diluted in PBS) to block Fc receptors before washing with PBS. Tumor cells harvested on following single CPMV dose were tested using the innate panel and were incubated at 4 °C in triplicate with the cocktail of zombie yellow viability, CD45, CD11b, CD11c, F4/80, CD86 and I-A/I-E antibodies diluted in PBS. Tumor cells harvested at 24 h following three doses of CPMV immunotherapy were tested using the adaptive panel and were incubated at 4 °C in triplicate with the cocktail of zombie yellow viability, CD45, CD3, CD25, CD4, CD8 and FOXP3 antibodies. Cells were washed twice with PBS and then fixed with 3% (*v*/*v*) paraformaldehyde for flow cytometry using an LSR II (BD Biosciences, San Jose, CA, USA). The data were analyzed using the FlowJo v8.6.3 software (Flow Jo, Ashland, OR, USA).

### 4.7. Immunohistochemistry and H&E-Staining

Brain tissues harvested from treated and untreated mice (*n* = 3) were fixed in 10% (*v*/*v*) buffered formalin, embedded in paraffin, sectioned at 6 μm, and mounted on Superfrost^®^ Plus slides (12-550-15, ThermoFisher Scientific). Sections were then hydrated through descending ethanol to water. Endogenous peroxidase activity was eliminated by incubation in 3% (*v*/*v*) H_2_O_2_ for 30 min prior to heat induced epitope retrieval (HIER). HIER was performed using a citrate based retrieval buffer, pH 6.1 (S1699, Dako, Santa Clara, CA, USA) for 10 min in a 96 °C water bath. The mouse antigen blocking kit (PK-2200, Vector laboratories, Burlingame, CA, USA) was utilized to reduce background staining according to manufacturer protocols for mouse derived antibodies. To reduce non-specific binding sections were incubated in 10% (*v*/*v*) normal goat serum (PCN5000, ThermoFisher Scientific) in Tris-buffered saline, (TBS; 50 mM Tris-HCl 150 mM NaCl, pH 7.6, Bio-Rad, 170-6435, Hercules, CA, USA) for 30 min prior to application of the primary antibody. Antibodies used in this study were mouse monoclonal antibody specific CD68 (ab201340, Abcam, Cambridge, UK) and rabbit monoclonal antibodies to IBA1 (ab178846, Abcam); CD45 (ab10558, Abcam) and Foxp3 (700914, Invitrogen, Camarillo, CA, USA). Immunohistochemistry was visualized via the peroxidase-anti-peroxidase method using 3,3′-diaminobenzidine (DAB) as a chromogen (TA-125-QHDX, ThermoFisher Scientific, Waltham, MA, USA). Serial sections were stained with hematoxylin and eosin to note the areas of tumor cell growth. Images were acquired with an Azio Scope A.1 (Zeiss, Thronwood, NY, USA) from three adjacent fields containing tumor. The immunoreactive intensity of positive cells were measured utilizing the Zeiss image analysis program (Axiovision Red 4.5, Zeiss) with background levels substracted from the stroma of the tumor. Statistical analysis was completed by ordinary one-way ANOVA using the Tukey’s multiple comparisons test on the GraphPad Prism software (GraphPad Software, San Diego, CA, USA).

## 5. Conclusions

In conclusion, our results illustrate that eCPMV-mediated modulation of the immunological landscape in the brain TME supports anti-tumor response in our murine model. With its non-pathogenic and non-replicating nature, ability to reverse the tumor immunosuppression and recruit immune effector cells, eCPMV nanoparticles offer a promising immunotherapy for glioma. In this study, we performed multiple intracranial treatments; in the future one may consider the development of slow-release formulations, or continuous low flow infusion to better control the immune response and alleviate the edema and complications associated with immunotherapy. Delivery of therapeutics to the brain is an active area of research that has evolved from biodegradable polymer implants [65,66] to more recent miniaturized implantable system MiNDS [67]. We have already developed and tested slow-release formulations for VLP vaccines [68], including in situ vaccines [69]. By formulating slow-release devices or implants incorporating VLPs, it will likely be possible to circumvent the need for repeated invasive administrations; thereby improving the translational potential of plant virus based immunotherapy for glioma.
